# The influence of serum uric acid on renal function in patients with calcium or uric acid stone: A population-based analysis

**DOI:** 10.1371/journal.pone.0182136

**Published:** 2017-07-31

**Authors:** Yoshimi Tanaka, Shingo Hatakeyama, Toshikazu Tanaka, Hayato Yamamoto, Takuma Narita, Itsuto Hamano, Teppei Matsumoto, Osamu Soma, Teppei Okamoto, Yuki Tobisawa, Tohru Yoneyama, Takahiro Yoneyama, Yasuhiro Hashimoto, Takuya Koie, Ippei Takahashi, Shigeyuki Nakaji, Yuriko Terayama, Tomihisa Funyu, Chikara Ohyama

**Affiliations:** 1 Department of Urology, Hirosaki University Graduate School of Medicine, Hirosaki, Japan; 2 Department of Advanced Transplant and Regenerative Medicine, Hirosaki University Graduate School of Medicine, Hirosaki, Japan; 3 Department of Social Medicine, Hirosaki University School of Medicine, Hirosaki, Japan; 4 Department of Urology, Oyokyo Kidney Research Institute, Hirosaki, Japan; Istituto Di Ricerche Farmacologiche Mario Negri, ITALY

## Abstract

**Objectives:**

To determine the influence of serum uric acid (UA) levels on renal impairment in patients with UA stone.

**Materials and methods:**

We retrospectively analyzed 463 patients with calcium oxalate and/or calcium phosphate stones (CaOx/CaP), and 139 patients with UA stones. The subjects were divided into the serum UA-high (UA ≥ 7.0 mg/dL) or the UA-low group (UA < 7.0 mg/dL). The control group comprised 3082 community-dwelling individuals that were pair-matched according to age, sex, body mass index, comorbidities, hemoglobin, serum albumin, and serum UA using propensity score matching. We compared renal function between controls and patients with UA stone (analysis 1), and between patients with CaOx/CaP and with UA stone (analysis 2). Logistic regression analysis was used to evaluate the impact of the hyperuricemia on the development of stage 3 and 3B chronic kidney disease (CKD) (analysis 3).

**Results:**

The renal function was significantly associated with serum UA levels in the controls and patients with CaOx/CaP and UA stones. In pair-matched subgroups, patients with UA stone had significantly lower renal function than the control subjects (analysis 1) and patients with CaOx/CaP stones (analysis 2) regardless of hyperuricemia. Multivariate logistic regression analysis revealed that patients with UA stone, CaOx/CaP, hyperuricemia, presence of cardiovascular disease, higher body mass index, older age and lower hemoglobin had significantly higher risk of stage 3 and 3B CKD (analysis 3).

**Conclusion:**

Patients with UA stones had significantly worse renal function than controls and CaOx/CaP patients regardless of hyperuricemia. Urolithiasis (CaOx/CaP and UA stone) and hyperuricemia had an association with impaired renal function. Our findings encourage clinicians to initiate intensive treatment and education approaches in patients with urolithiasis and/or hyperuricemia in order to prevent the progression of renal impairment.

## Introduction

The prevalence of urolithiasis has been increasing in Japan, similar to other developed countries [1]. Urinary stones can be composed of different substances, including calcium oxalate (CaOx), calcium phosphate (CaP), and uric acid (UA). The prevalence of UA stones varies according to geographical region, with a prevalence of 5–10% in the United States, 17–25% in Germany, 4% in Sweden, and up to 40% in Israel. In Japan, the prevalence of UA stones is an estimated 13.8% in men and 3.8% in women [2, 3]. Although UA stones are not a predominant composition of urolithiasis, patients with UA stones have significantly worse renal function compared with CaOx or CaP stones [4].

Urolithiasis is reported to be associated with metabolic syndrome (MetS) [5]. MetS is associated with hypertension, obesity, high cholesterol, hyperuricemia, type 2 diabetes, atherosclerotic cardiovascular disease (CVD), and chronic kidney disease (CKD) [6–9]. Patients with MetS have a higher prevalence of UA stones compared with other types of urinary stones [10]. Meanwhile, urolithiasis often presents in patients with hyperuricemia. Hyperuricemia is considered an independent risk factor for renal impairment in renal cell carcinoma patients after unilateral nephrectomy [11], renal transplant recipients [12, 13], and in the general population as well [14, 15]. Although the precise relationship between hyperuricemia and urolithiasis remains unclear, hyperuricemia-associated symptoms such as hyperuricosuria and acidic urine are well-established contributors to the formation of UA stones [2, 16, 17]. These findings indicate that both hyperuricemia and UA stones are potential risk factors for CKD [10, 18]. However, the influence of serum UA levels on renal impairment in patients with urolithiasis is not well known. For example, although hyperuricemia is a risk factor for CaOx/CaP stones, UA stone patients do not always present with hyperuricemia. Furthermore, urolithiasis patients with impaired renal function do not always present with hyperuricemia. Therefore, we sought to determine the influence of hyperuricemia on the development of chronic kidney disease in patients with urolithiasis. In the present study, we retrospectively analyzed the influence of serum UA levels (≥ 7.0 mg/dL vs. < 7.0 mg/dL) on impaired renal function in patients with urolithiasis and control individuals from a community-dwelling population.

## Materials and methods

### Ethics statement

The study was conducted in accordance with the ethical standards of the Declaration of Helsinki and was approved by the Ethics Committee of Hirosaki University Graduate School of Medicine (authorization number 2016–225). For this type of retrospective study, formal patient consent is not required. The cross-sectional data collection from the Iwaki Health Promotion Project was approved by the Ethics Committee of Hirosaki University School of Medicine (authorization number 2014–015), and all of the subjects provided written informed consent before participating in the study. This study was registered as a clinical trial UMIN000022962.

### Patient selection

Between January 2010 and September 2015, 1319 patients with urolithiasis were treated at the Oyokyo Kidney Research Institute and Hirosaki University Hospital. Patients with infection stones, and patients lacking sufficient clinical data regarding urinary stones and blood examinations were excluded from the study. Ultimately, 602 patients who underwent laboratory testing to evaluate renal function, serum UA levels, and lipid metabolism were included in this retrospective study. Among them, 463 patients with CaOx/CaP and 139 patients with UA stones were identified. The patients were divided into two groups according to serum UA levels: the UA-high group with hyperuricemia (serum UA ≥ 7.0 mg/dL) or the UA-low group with normal UA levels (serum UA < 7.0 mg/dL) groups. The 3082 community-dwelling control subjects (Ctrl) were selected from the Iwaki Health Promotion Project. The Iwaki Health Promotion project was a comprehensive study for clarification of etiology for lifestyle-related diseases such as hypertension, cardiovascular diseases, atherosclerosis, chronic renal failure, osteoporosis, arthritis, asthma, cancer, chronic liver disease or cirrhosis, chronic obstructive pulmonary disease, type 2 diabetes, obesity, metabolic syndrome, dementia, Alzheimer’s disease and depression. It was planned to prevent lifestyle-related diseases and promote health to extend the life span of residents of Hirosaki city (Iwaki district), a northern part of Japan in collaboration with Hirosaki University, Hirosaki City, and Aomori Prefecture general screening center. This project conducted comprehensive screening of general health status including comprehensive biological and physical examinations.

### Evaluation of variables

The analyzed pre-treatment variables were age, sex, body mass index, history of hypertension (HTN), blood pressure, diabetes mellitus (DM), cardiovascular disease (CVD), renal function, hemoglobin, serum albumin (Alb), serum UA, dyslipidemia (total cholesterol [Tcho] and triglyceride [TG]) and type of stones (Calcium oxalate [CaOx], Calcium phosphate [CaP], CaOx/CaP mixed, UA containing stone, magnesium ammonium phosphate [struvite stone, NH_4_MgPO_4_], and cystin). CVD was defined as a positive history of cardiac surgery, angina, myocardial infarction, or stroke or taking any cardiotonic agents or coronary vasodilators. Estimated glomerular filtration rate (eGFR) was used as an index of renal function, and it was calculated using the Modification of Diet in Renal Disease equation for Japanese patients [19]. Stage 3 and 3B CKD was defined as eGFR < 60 and <45 mL/min/1.73 m^2^, respectively.

### Outcome measurements

To adjust the background differences, they were matched to the study patients using propensity-score matching [20]. We compared background characteristics, eGFR, and number of stage 3 CKD individuals between control individuals and patients with UA stones. To evaluate the impact of the UA stone on stage 3 and 3B CKD, multivariate logistic regression analyses were performed including the 3082 control subjects and the 602 urolithiasis patients (CaOx/CaP: n = 463 and UA stone: n = 139).

### Statistical analysis

Statistical analyses were conducted using SPSS v. 24.0 (IBM Corporation, Armonk, NY, USA) and GraphPad Prism v. 5.03 (GraphPad Software, San Diego, CA, USA). Categorical variables were presented as percentages and compared using Fisher’s exact test or Chi-square test. Quantitative data were expressed as the mean ± standard deviation. Differences between groups were compared using Student’s *t*-test (data with a normal distribution) or Mann-Whitney U-test (data with a non-normal distribution). The correlation between two parameters was analyzed using Spearman’s correlation coefficient. Probability (*P*) values < 0.05 were considered statistically significant.

To select the appropriate control subjects from the 3082 community-dwelling individuals, we compared renal function between control subjects and patients with UA stones (analysis 1), and between CaOx/CaP and patients with UA stones (analysis 2) using propensity score matching as previously described [21]. Propensity scores were calculated using logistic analysis, and they accounted for age, sex, body mass index, comorbidities (HTN, DM and CVD), hyperlipidemia (Tcho > 220 mg/dL or TG >150 mg/dL), hemoglobin, serum Alb, and serum UA levels. Two healthy subjects and one UA stone patient with a score within 0.03 points of one another were selected as a paired group. We performed a multivariate logistic regression analysis in the 3082 control subjects and the 602 urolithiasis patients to evaluate potential risk factors for the development of stage 3 and 3B CKD. Variables included in the logistic regression analysis for the development of stage 3 and 3B CKD were age, sex, body mass index, HTN, DM, CVD, hemoglobin, hyperlipidemia, serum Alb and hyperuricemia (UA ≥ 7.0 mg/dL). The odds ratios (ORs) with 95% confidence intervals (CIs) associated with each parameter were calculated after adjusting for potentially confounding factors.

## Results

### Clinical characteristic of subjects in the present study

**Fig 1:**
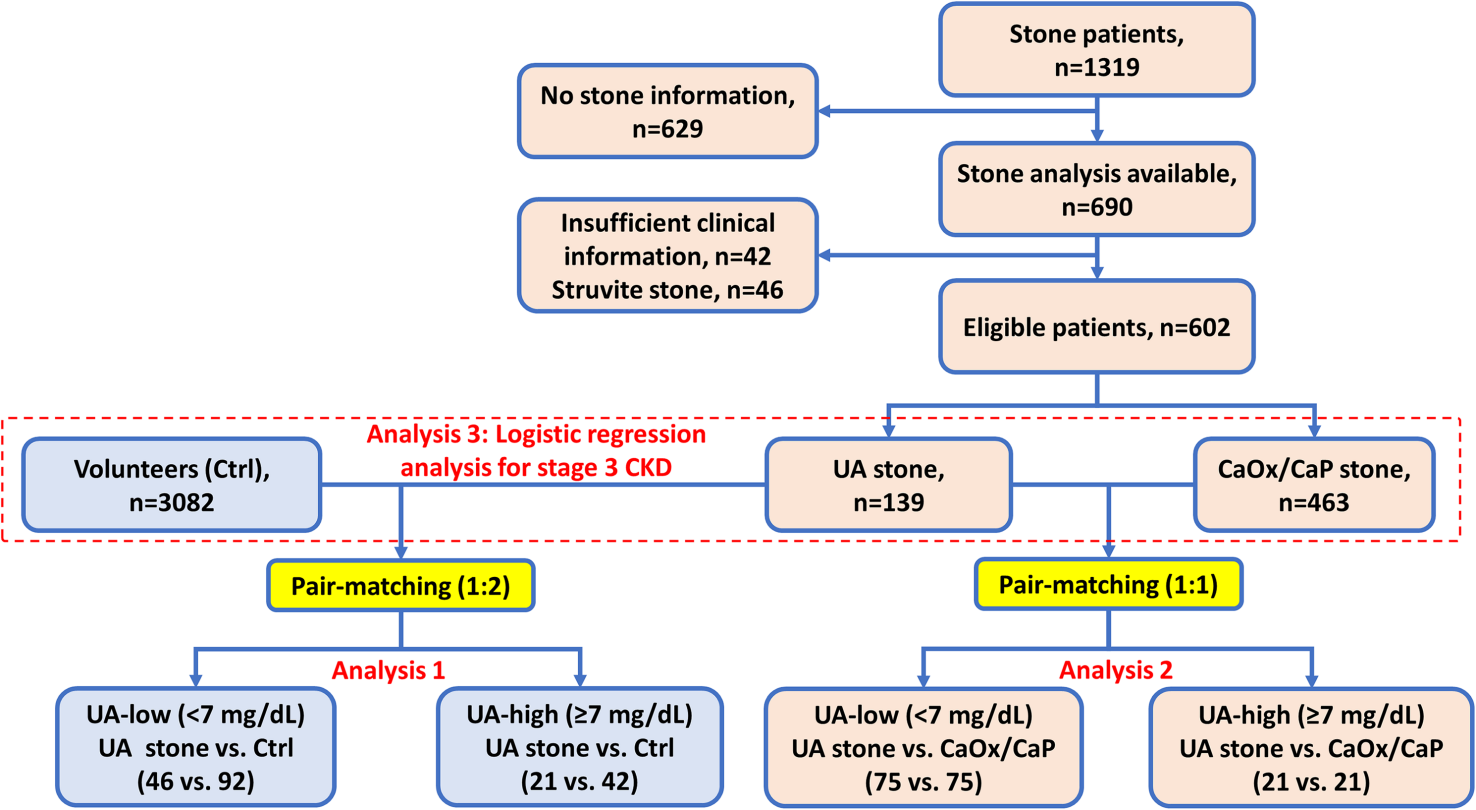
Patient selection and classification. Eligible patients with urinary stones and control subjects selected from the database. We excluded 629 patients without stone analysis, 42 patients without complete clinical data, and 46 struvite stones. The remaining 602 urinary stone patients included in the study. The subjects were divided into two groups according to UA levels: the UA-high group with hyperuricemia (UA ≥ 7.0 mg/dL) or the UA-low group with normal UA levels (UA < 7.0 mg/dL). The control subjects and stone patients were pair-matched according to age, sex, body mass index, comorbidities (HTN, DM and CVD), hyperlipidemia hemoglobin, serum Alb, and serum UA levels using propensity score matching.

**Fig 2:**
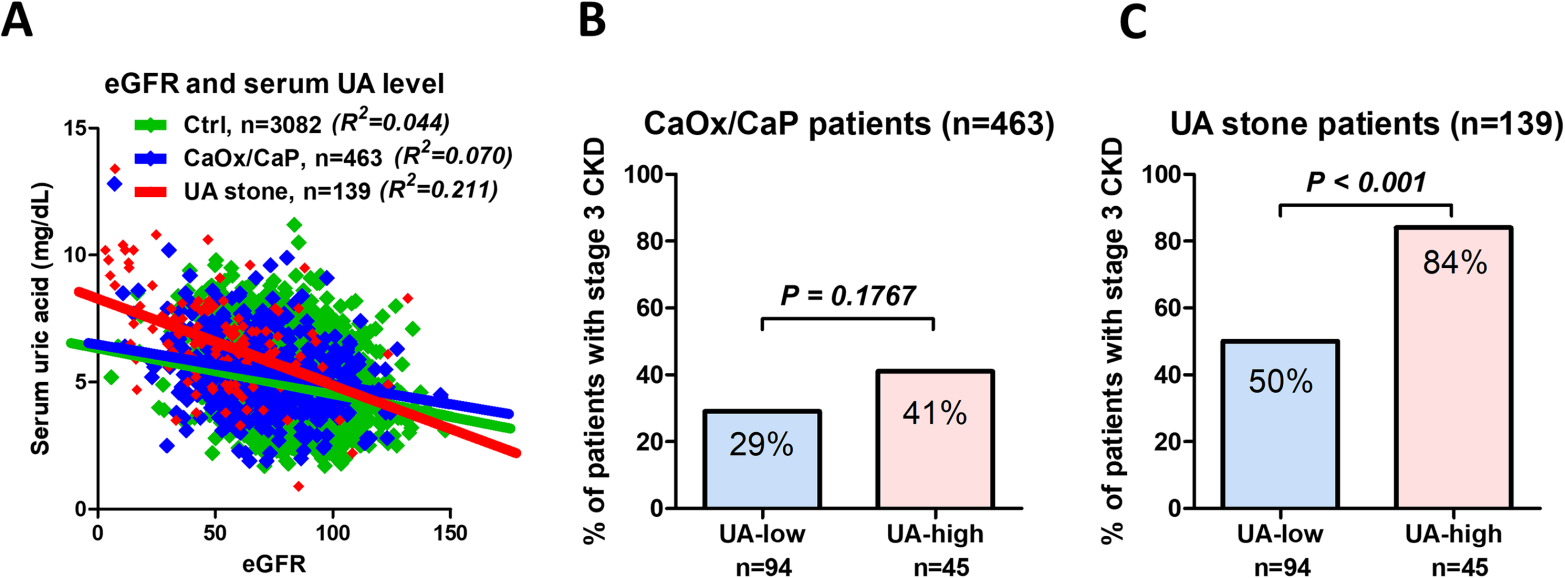
The relationship between serum UA level and eGFR. The correlation between eGFR and serum UA levels were significant in Ctrl, CaOx/CaP, and UA stone patients. R^2^ values showed higher correlation in UA stone patients (R^2^ = 0.211, *P <* 0.001) than that in the Ctrl (R^2^ = 0.044, *P <* 0.001) or in the CaOx/CaP or (R^2^ = 0.070, *P <* 0.001) (A). The number of CaOx/CaP patients with stage 3 CKD was not significantly different between the UA-low and UA-high (B). The number of UA stone patients with stage 3 CKD was significantly higher in the patients with UA-high than with UA-low group (C).

In total, we evaluated 4401 subjects for analysis including 1319 patients with urolithiasis and the control group comprised 3082 community-dwelling individuals. From 1319 patients, we selected eligible 463 patients with CaOx/CaP and 139 patients with UA stone (Fig 1). Clinical characteristic of subjects in the present study were shown in Table 1. Patient background was significantly different between the UA stone patients and the patients with CaOx/CaP stones in age, sex, body mass index, HTN, DM, CVD, hemoglobin, serum Alb, serum UA, hyperuricemia, triglyceride, and stage 3 CKD. The correlation between eGFR and serum UA levels were significant. R^2^ values showed higher correlation in UA stone patients (R^2^ = 0.211, *P <* 0.001) than that in the Ctrl (R^2^ = 0.044, *P <* 0.001) or in the CaOx/CaP or (R^2^ = 0.070, *P <* 0.001) (Fig 2A).

**Table 1:** Clinical characteristic of the population in the present study.

			Eligible patients	
	Ctrl	Stone former	Non-UA stone	UA stone
Number, n =	3082	1319	463	139
Age*, years (IQR)	57 (44–66)	57 (46–68)	59 (48–68)	71 (61–79)
Sex*, male, n =	1177 (38%)	858 (65%)	160 (35%)	123 (88%)
Body mass index* (kg/m2)	20.6 (19.8–21.5)	21.1 (18.9–23.4)	22.5 (21–25.4)	24.3 (21.8–26.6)
Blood pressure (mmHg)				
Systolic	127 (114–140)	130 (120–140)	130 (120–140)	133 (122–150)
Diastolic	76 (68–85)	78 (70–80)	78 (70–80)	80 (70–81)
Hypertension* (HTN), n =	1107 (36%)	459 (35%)	160 (35%)	107 (77%)
Diabetes mellitus* (DM), n =	234 (8%)	215 (16%)	74 (16%)	48 (35%)
Cardiovascular disease (CVD), n =	247 (8.0%)	127 (10%)	40 (9%)	32 (23%)
Hemoglobin* (Hb) (g/dL)	13.6 (12.7–14.8)	13.7 (12.5–15.1)	13.8 (12.7–15.1)	13.3 (11.8–14.6)
Serum Albumin* (Alb) (g/dL)	4.5 (4.3–4.7)	4.2 (3.9–4.4)	4.2 (3.9–4.4)	4.0 (3.7–4.2)
Serum uric acid* (UA) (mg/dL)	4.7 (3.9–5.8)	5.4 (4.5–6.5)	5.3 (4.3–6.3)	6.2 (5.3–7.5)
Hyperuricemia (UA ≥ 7.0 mg/dL), n =	271 (8.8%)	155/885 (18%)	63 (14%)	45 (32%)
UA value unknown, n =	0	434	0	0
eGFR (mL/min/1.73m^2^)	79.3 (69.8–88.9)	68.1 (57.7–84.1)	71.2 (55.5–86.4)	54.4 (36.0–67.4)
Stage 3 CKD, n =	257 (8.3%)	329/885 (37%)	143 (31%)	85 (61%)
eGFR value unknown, n =	0	434	0	0
Total cholesterol (Tcho) (mg/dL)	203 (181–226)	190 (166–218)	190 (169–216)	189 (162–220)
Triglyceride (TG) (mg/dL)	79 (57–113)	108 (77–153)	106 (76–145)	112 (830177)
Dyslipidemia* (Tcho >220, or TG >140 mg/dL), n =	2090 (68%)	383/986 (39%)	189 (41%)	65 (47%)
Tcho or TG value unknown, n =	0	333	0	0
Stone information available, n =		690 (52%)	463	139
Type of stone, n =				
Calcium oxalate (CaOx)		277 (21%)	259 (56%)	0
Calcium phosphate (CaP)		31 (2.4%)	28 (6.0%)	0
CaOx/CaP mixed		185 (14%)	176 (38%)	0
Uric acid containing stone		147 (11%)	0	139
Ammonium magnesium phosphate (NH4MgPO4)		46 (3.5%)	0	0
Cystin		4 (0.3%)	0	0
Stone information unavailable, n =		629 (48%)	0	0

*, applied for propensity score-matching

The number of CaOx/CaP patients with stage 3 CKD were not significantly different between the UA-low and UA-high (Fig 2B). The number of UA stone patients with stage 3 CKD were significantly higher in the patients with UA-high than with UA-low group (Fig 2C)

### Comparison of control subjects and UA stone patients (analysis 1)

**Fig 3:**
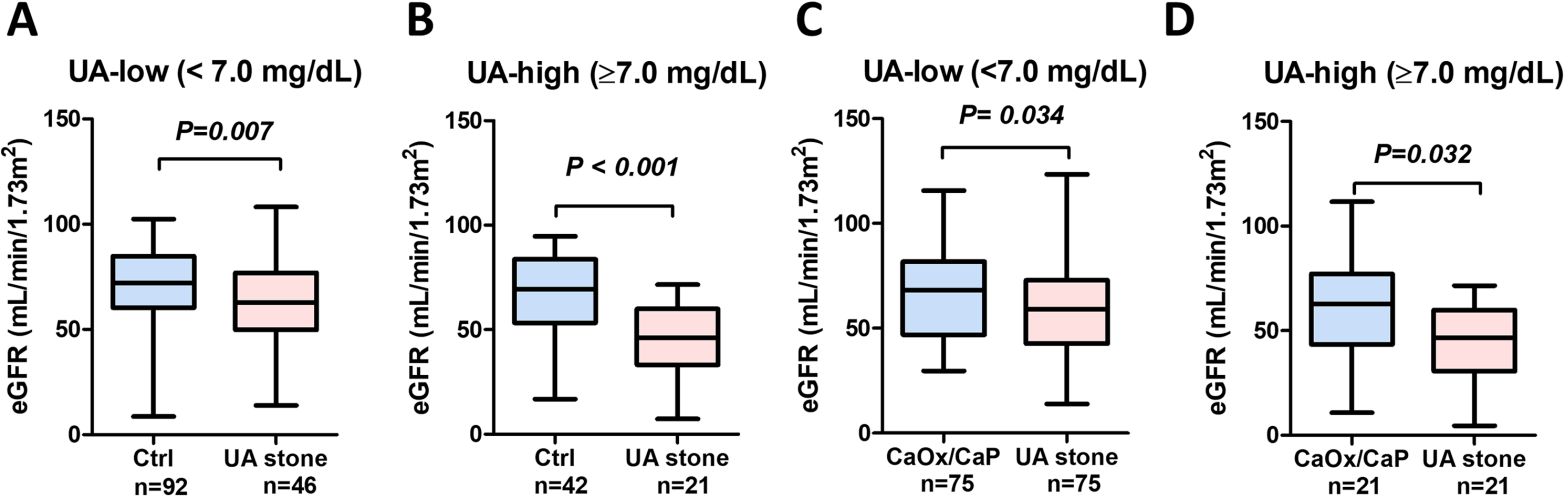
The prevalence of renal impairment in pair-matched subjects stratified serum UA level. In the UA-low group (serum UA < 7.0 mg/dL), eGFR was significantly lower in patients with UA stones compared with control subjects (A). The prevalence of stage 3 CKD was significantly greater in UA stone patients (48%) compared with control subjects (24%) (*P* = 0.005). In the UA-high group (serum UA ≥ 7.0 mg/dL), eGFR was significantly lower in patients with UA stones compared with control subjects (B). The prevalence of stage 3 CKD was significantly greater in UA stone patients (76%) compared with control subjects (45%) (*P* = 0.020). In the UA-low group, eGFR was significantly lower in patients with UA stones compared with CaOx/CaP (*P* = 0.034) (C). The prevalence of stage 3 CKD was not significantly different between in patients with UA stone (53%) and CaOx/CaP (40%) (*P* = 0.414). In the UA-high group, eGFR was significantly lower in patients with UA stones compared with CaOx/CaP (*P* = 0.032) (D). The prevalence of stage 3 CKD was significantly greater in UA stone patients (76%) compared with CaOx/CaP (38%) (*P* = 0.028).

The UA-low group comprised 92 control subjects and pair-matched 46 patients with UA stones. There were no statistically significant differences in background between UA stone patients and control subjects (Table 2). However, eGFR was significantly lower in UA stone patients (62 ± 21 mL/min/1.73m^2^) compared with control subjects (71 ± 17 mL/min/1.73m^2^) (*P* = 0.007) (Fig 3A). The prevalence of stage 3 CKD was significantly greater in UA stone patients (48%) compared with control subjects (24%) (*P* = 0.005)

**Table 2:** Clinical characteristic of pair-matched subjects (analysis 1).

	UA-low (<7 mg/dL) group			UA-high (≥7 mg/dL) group		
Pair-matching (2:1)	Ctrl	UA stone	*P value*	Ctrl	UA stone	*P value*
n	92	46		42	21	
Age^**†**^, years	65 ± 12	66 ± 14	*0*.*678*	64 ± 13	63 ± 17	*0*.*838*
Sex, male, n =	75 (82%)	37 (80%)	*0*.*880*	39 (93%)	18 (86%)	*0*.*423*
Body mass index^**†**^(kg/m2)	23 ± 2.5	23 ± 3.4	*0*.*756*	24 ± 1.7	24 ± 3.0	*0*.*583*
Hypertension (HTN), n =	61 (66%)	30 (65%)	*0*.*900*	27 (64%)	12 (57%)	*0*.*596*
Diabetes mellitus (DM), n =	25 (27%)	13 (28%)	*0*.*895*	36 (29%)	17 (27%)	*0*.*574*
Cardiovascular disease (CVD), n =	16 (17%)	7 (15%)	*0*.*745*	9 (21%)	2 (10%)	*0*.*200*
Hemoglobin^**†**^ (Hb) (g/dL)	13.8 ± 1.4	13.8 ± 1.7	*0*.*995*	14.2 ± 1.4	13.8 ± 1.1	*0*.*184*
Serum Albumin^**†**^ (Alb) (g/dL)	4.1 ± 0.3	4.1 ± 0.3	*0*.*971*	4.2 ± 0.3	4.2 ± 0.3	*0*.*980*
Serum uric acid^**†**^ (UA) (mg/dL)	5.8 ± 0.9	5.5 ± 1.1	*0*.*119*	8.1 ± 0.9	8.2 ± 1.1	*0*.*729*
Total cholesterol^**†**^ (Tcho) (mg/dL)	197 ± 32.4	206 ± 43.3	*0*.*251*	217 ± 39	216 ± 36	*0*.*942*
Triglyceride^**†**^ (TG) (mg/dL)	139 ± 165	131 ± 75.5	*0*.*709*	193 ± 211	182 ± 135	*0*.*800*
Type of stone, n =						
Pure-UA stone		25 (54%)			16 (76%)	
UA mixed stone		21 (46%)			5 (24%)	

^**†**^, mean ± standard deviation

The UA-high group comprised 21 patients with UA stones and 42 pair-matched control subjects. There were no statistically significant differences in background between UA stone patients and control subjects (Table 2). However, eGFR was significantly lower in UA stone patients (47 ± 26 mL/min/1.73m^2^) compared with control subjects (66 ± 19 mL/min/1.73m^2^) (*P* < 0.001) (Fig 3B). The prevalence of stage 3 CKD was significantly greater in UA stone patients (76%) compared with control subjects (45%) (*P* = 0.020).

### Comparison of CaOx/CaP and UA stone patients (analysis 2)

The UA-low group comprised pair-matched 75 patients with CaOx/CaP and 75 patients with UA stones. There were no statistically significant differences in background between UA stone patients and CaOx/CaP patients (Table 3). The eGFR was significantly lower in patients with UA stones (59 ± 21 mL/min/1.73m^2^) compared with CaOx/CaP (66 ± 20 mL/min/1.73m^2^) (*P* = 0.034) (Fig 3C). However, the prevalence of stage 3 CKD was not significantly different between patients with CaOx/CaP (40%) and UA stone (53%) (*P* = 0.414).

**Table 3:** Clinical characteristic of pair-matched subjects (analysis 2).

	UA-low (<7 mg/dL) group			UA-high (≥7 mg/dL) group		
Pair-matching (1:1)	CaOx/CaP	UA stone	*P value*	CaOx/CaP	UA stone	*P value*
n	75	75		21	21	
Age^**†**^, years	68 ± 13	68 ± 13	*0*.*910*	59 ± 12	60 ± 17	*0*.*804*
Sex, male, n =	60 (80%)	64 (85%)	*0*.*388*	19 (90%)	17 (81%)	*0*.*663*
Body mass index^**†**^(kg/m2)	25 ± 4.9	24 ± 3.7	*0*.*204*	26 ± 4.1	24 ± 3.8	*0*.*195*
Hypertension (HTN), n =	44 (59%)	55 (73%)	*0*.*058*	9 (43%)	13 (62%)	*0*.*217*
Diabetes mellitus (DM), n =	18 (24%)	24 (32%)	*0*.*275*	1 (5%)	5 (24%)	*0*.*184*
Cardiovascular disease (CVD), n =	10 (13%)	15 (20%)	*0*.*273*	2 (10%)	3 (18%)	*1*.*000*
Hemoglobin^**†**^ (Hb) (g/dL)	13.4 ± 1.8	13.6 ± 1.9	*0*.*589*	13.8 ± 2.0	13.6 ± 1.4	*0*.*714*
Serum Albumin^**†**^ (Alb) (g/dL)	3.9 ± 0.5	4.0 ± 0.3	*0*.*123*	4.1 ± 0.5	4.1 ± 0.4	*0*.*873*
Serum uric acid^**†**^ (UA) (mg/dL)	5.3 ± 1.0	5.4 ± 1.1	*0*.*334*	8.0 ± 0.8	8.1 ± 1.0	*0*.*698*
Total cholesterol^**†**^ (Tcho) (mg/dL)	186 ± 36	195 ± 38	*0*.*128*	198 ± 35	209 ± 44	*0*.*362*
Triglyceride^**†**^ (TG) (mg/dL)	128 ± 80	136 ± 84	*0*.*564*	158 ± 70	190 ± 135	*0*.*353*
Type of stone, n =						
Pure-UA stone		43 (57%)			10 (48%)	
UA mixed stone		32 (43%)			11 (52%)	

^**†**^, mean ± standard deviation

The UA-high group comprised 21 patients with CaOx/CaP and pair-matched 21 patients with UA stones. There were no statistically significant differences in background between UA stone patients and control subjects (Table 3). The eGFR was significantly lower in patients with UA stones (47 ± 27 mL/min/1.73m^2^) compared with CaOx/CaP (60 ± 25 mL/min/1.73m^2^) (*P* = 0.032) (Fig 3D). The prevalence of stage 3 CKD was significantly greater in patients with UA stones (76%) compared with CaOx/CaP (38%) (*P* = 0.028).

### Independent risk factors for development of stage 3 and 3B CKD (analysis 3)

**Fig 4:**
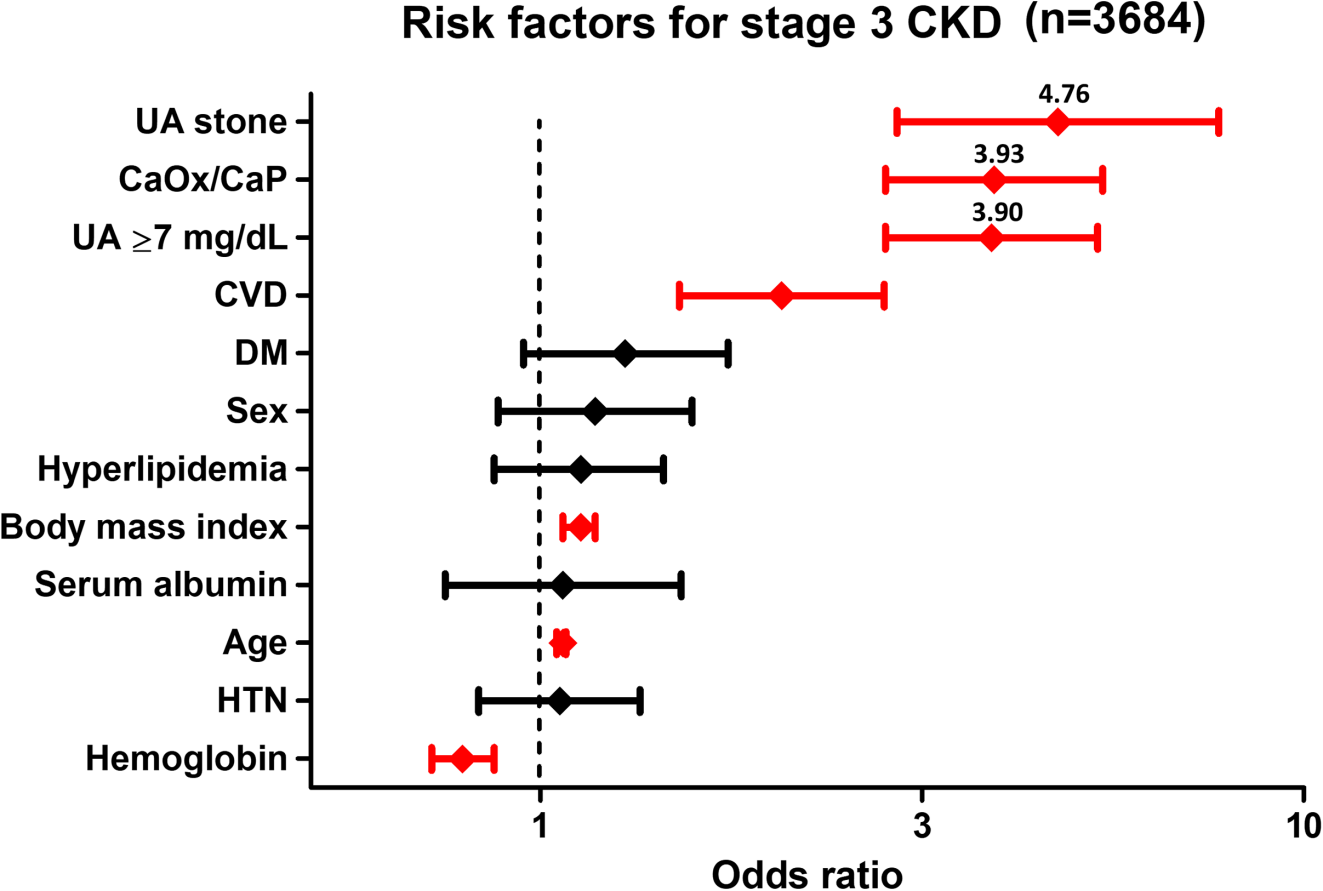
Multivariate logistic regression analysis for stage 3 CKD. Independent risk factors for development of stage 3 CKD in the 3082 control subjects and 602 stone patients were evaluated using multivariate logistic regression analysis including 12 variables. UA stone, CaOx/CaP, hyperuricemia, presence of CVD, body mass index, age and hemoglobin were selected as independent risk factors for stage 3 CKD.

**Fig 5:**
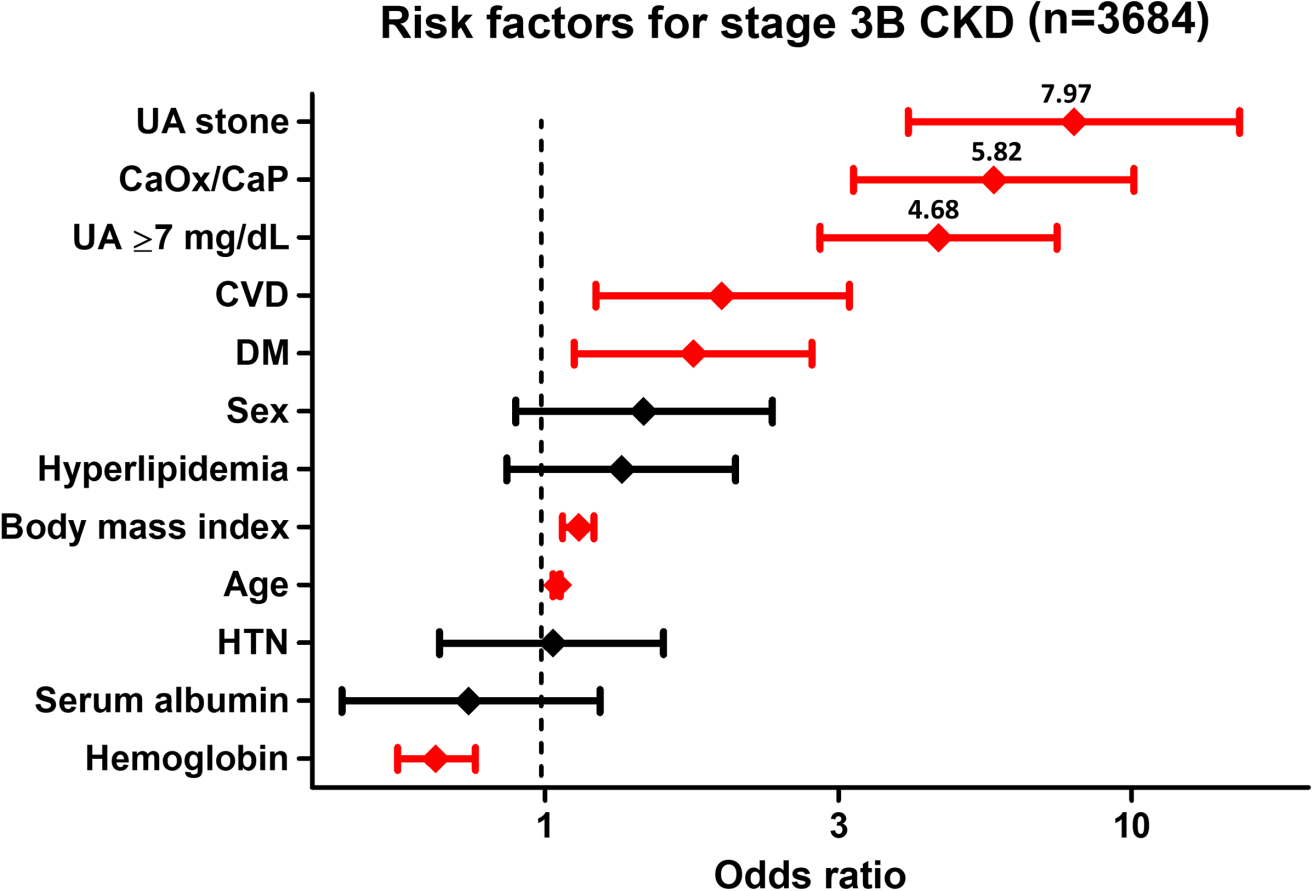
Multivariate logistic regression analysis for stage 3B CKD. Age, body mass index, presence of CVD, DM, hemoglobin, hyperuricemia, CaOx/CaP, and UA stone were selected as independent risk factors for stage 3B CKD.

Independent risk factors for development of stage 3 and 3B CKD in the 3082 control subjects and 602 stone patients was evaluated using uni- and multivariate logistic regression analyses including 12 variables. In univariate analysis, all variables were selected as independent risk factors for development of stage 3 and 3B CKD. UA stone (OR: 4.76, 95% CI: 2.93–4.74), CaOx/CaP stone (OR: 3.93, 95% CI: 2.83–3.43), and hyperuricemia (OR: 3.90, 95% CI: 2.83–3.37) persisted as an independent risk factors for stage 3 CKD after multivariate logistic regression analysis accounting for the strong confounders such as age, body mass index, CVD, and hemoglobin (Fig 4, Table 4). Similarly, age, body mass index, presence of CVD, DM, hemoglobin, hyperuricemia (OR: 4.68, 95% CI: 2.94–7.45), CaOx/CaP (OR: 5.82, 95% CI: 3.35–10.1), and UA stone (OR: 7.97, 95% CI: 4.16–15.3) were selected as independent risk factors for development of stage 3B CKD (Fig 5, Table 5). Minimal data are available in the S1 Dataset (MS Excel file)

**Table 4:** Independent risk factors for stage 3 CKD by uni- and multivariate logistic regression analysis (analysis 3).

		Univariate			Multivariate		
Variable	Risk factor	*P value*	OR	95%CI	*P value*	OR	95%CI
Age	Continuous	*<0*.*001*	1.08	1.07–1.09	*<0*.*001*	1.07	1.05–1.08
Sex	Male	*<0*.*001*	1.67	1.38–2.03	*0*.*282*	1.18	0.88–1.58
Body mass index	Continuous	*<0*.*001*	1.30	1.26–1.35	*<0*.*001*	1.13	1.07–1.18
HTN	Positive	*<0*.*001*	2.60	2.14–3.16	*0*.*636*	1.06	0.83–1.35
CVD	Positive	*<0*.*001*	4.27	3.31–5.50	*<0*.*001*	2.07	1.52–2.82
DM	Positive	*<0*.*001*	3.25	2.52–4.17	*0*.*107*	1.29	0.95–1.76
Hyperlipidemia	Positive	0.010	0.77	0.64–0.94	*0*.*359*	1.13	0.87–1.45
Hemoglobin	Continuous	*<0*.*001*	0.81	0.77–0.86	*<0*.*001*	0.79	0.72–0.87
Serum albumin	Continuous	*<0*.*001*	0.16	0.12–0.20	*0*.*702*	1.07	0.75–1.53
Serum UA level	≥7 mg/dL	*<0*.*001*	4.08	3.21–5.18	*<0*.*001*	3.90	2.83–3.37
Type of stones	CaOx/CaP	*<0*.*001*	3.76	3.00–4.72	*<0*.*001*	3.93	2.83–3.45
	UA stone	*<0*.*001*	12.4	8.66–17.7	*<0*.*001*	4.76	2.93–4.74

**Table 5:** Independent risk factors for stage 3B CKD by uni- and multivariate logistic regression analysis (analysis 3).

		Univariate			Multivariate		
Variable	Risk factor	*P value*	OR	95%CI	*P value*	OR	95%CI
Age	Continuous	*<0*.*001*	1.08	1.07–1.10	*<0*.*001*	1.05	1.03–1.06
Sex	Male	*<0*.*001*	2.36	1.68–3.33	*0*.*133*	1.47	0.89–2.44
Body mass index	Continuous	*<0*.*001*	1.30	1.24–1.37	*<0*.*001*	1.14	1.07–1.21
HTN	Positive	*<0*.*001*	3.00	2.13–4.22	*0*.*914*	1.03	0.66–1.59
CVD	Positive	*<0*.*001*	4.24	2.88–6.24	*0*.*006*	2.00	1.22–3.30
DM	Positive	*<0*.*001*	4.46	3.07–6.47	*0*.*015*	1.79	1.12–2.85
Hyperlipidemia	Positive	*0*.*001*	0.56	0.40–0.78	*0*.*194*	1.35	0.86–2.11
Hemoglobin	Continuous	*<0*.*001*	0.66	0.60–0.73	*<0*.*001*	0.65	0.56–0.76
Serum albumin	Continuous	*<0*.*001*	0.09	0.06–0.13	*0*.*252*	0.74	0.45–1.24
Serum UA level	≥7 mg/dL	*<0*.*001*	6.31	4.44–8.97	*<0*.*001*	4.68	2.94–7.45
Type of stones	CaOx/CaP	*<0*.*001*	4.49	3.16–6.38	*<0*.*001*	5.82	3.35–10.1
	UA stone	*<0*.*001*	21.9	14.7–32.7	*<0*.*001*	7.97	4.16–15.3

## Discussion

Hyperuricemia is a significant risk factor for urolithiasis and CKD [22], but it is not always associated with UA stones. Although relationship between hyperuricemia and CKD in patients with urolithiasis has been suggested [16, 22], identifying an independent relationship among hyperuricemia, CKD and UA stone was challenging [23, 24]. In the present study, we investigated the impact of hyperuricemia on the risk of CKD in patients with UA stones. To this end, we conducted a comparative analysis of UA stone patients and pair-matched controls (volunteers without urolithiasis) and CaOx/CaP patients in a group of individuals with hyperuricemia and a group of individuals without hyperuricemia. We found that patients with UA stones had significantly worse renal function than controls and CaOx/CaP patients regardless of hyperuricemia. To adjust the potential confounders for CKD, we used a multivariate logistic regression analysis to evaluate the influence of UA stone on CKD. Our results demonstrated that CaOx/CaP and hyperuricemia had a similar impact (OR: 3.93 and 3.90, respectively), and UA stones had a higher impact (OR: 4.76) on stage 3 CKD. These results suggested the impact of UA stone on renal function seems to be higher than the one of serum UA levels. However, it is difficult to conclude that whether hyperuricemia or UA stone has a more detrimental effect on renal impairment because of overlapped range of wide 95%CI (2.93–4.74). At least, we should recognize that urolithiasis and hyperuricemia may have equivale impact on renal impairment from our observations.

Although hyperuricemia is a well-known risk factor for urolithiasis and renal impairment [10, 18], the etiologic relationships between hyperuricemia, UA stone, and CKD are complex. Chronic inflammation caused by MetS in patients with UA stone may be a possible explanation. The previous report indicated that patients with UA stones have chronic, low grade, and systemic inflammatory diseases [25]. In addition, several studies have addressed the clinical significance of MetS in patients with urolithiasis and CKD [9, 10, 14], especially in UA stones [1, 5, 14]. Another study reported that patients with UA stones had significantly higher rates of aortic calcification [26], which is a surrogate marker of arterial degradation. Furthermore, aortic calcification is directly correlated with CKD severity in renal transplant recipients [27], renal cell carcinoma patients who underwent radical nephrectomy [28] and urolithiasis patients [29], suggesting a potential surrogate marker for diminished renal reserve capacity. Although the mechanisms by which aortic calcification might influence glomerular microcapillary degeneration remain unclear, it is not hard to anticipate that vascular damage occurs first and more severely in small vessels such as afferent arterioles and glomeruli. Therefore, chronic inflammatory disease might play a key role in arterial degradation, thereby promoting the deterioration of renal function. However, the precise mechanism underlying the contribution of chronic inflammatory disease to renal impairment remains unclear. Further studies are necessary to address the detailed association between Mets, chronic inflammation, aortic calcification, UA stone, and CKD.

In the present study, we found that an UA stone patient have impaired renal function even with a normal serum UA level (Fig 3A and 3C). Although the reason remains unclear why UA stone patients with normal serum UA levels have impaired renal function, one possible reason for the renal impairment is persistent urine acidity caused by impaired buffering via defective urinary ammonium excretion. Ammonium is an important urinary buffer, and renal ammonium production and excretion are regulated by surrounding acid-base environment [30]. Impaired ammonium excretion is associated with insulin resistance, obesity, MetS and UA stone [31–33]. These results suggested that impaired ammonium excretion, which potentially linked to insulin-resistant state and MetS, lead the impaired renal function in UA stone patients. However, precise mechanisms need further investigation.

Chronic asymptomatic hyperuricemia (even in the normal ranges) may promote development of CKD and formation of UA stones. However, the lack of biomarkers for UA accumulation within the body remains an unmet need in patients with hyperuricemia. As efforts to improve chronic asymptomatic hyperuricemia are applied to patients with gout, this might be also a valuable therapeutic approach for patients with a combination of UA stones, insulin resistance, and MetS [34]. Optimal treatment of chronic asymptomatic hyperuricemia requires long-term reductions in serum UA levels. Urate-lowering agents used to treat renal impairment, including the xanthine oxidase inhibitors allopurinol, oxipurinol, and febuxostat, might also prevent CKD. A meta-analysis demonstrated that allopurinol and febuxostat might slow the progression of CKD; however, the results were inconclusive due to the small size of the studies analyzed [35, 36]. There is currently no definitive evidence to support the recommendation of urate-lowering agents for asymptomatic hyperuricemia in patients with CKD and urolithiasis. Large scale, randomized, and placebo-controlled trials are required to assess the effect of these agents in patients with CKD and urolithiasis.

Several limitations in this study should be noted. The limited sample size and retrospective study design prevented us from making definitive conclusions regarding the effect of serum UA levels on renal impairment in patients with UA stones. Furthermore, we were unable to control selection bias and other potentially confounding factors in UA stone patients or control subjects, despite the use of controlled matching methods. Our results did not account for some other factors known to influence renal function, including purine diet, the use of niacin, the amount of alcohol consumed or the amount of high fructose containing beverages, cigarette smoking, blood pressure control, background medications, proteinuria, and hydronephrosis. A history of urinary tract infections and/or administration of agents that are potentially renoprotective, or may alter plasma or urine uric acid excretion also may have an influence on renal function. The use of the Modification of Diet in Renal Disease equation for Japanese patients and using a cut-off of < 60 mL/min/1.73m^2^ to define CKD was also the limitation of the present study. Because the definition of CKD is the presence of GFR < 60 mL/min/1.73m^2^ for 3 months, eGFR evaluation at one point in time does not allow for conclusions regarding whether there is CKD or not. Even though the OR for UA stones was higher than hyperuricemia, it also had the widest 95%CI (2.93–4.74). Therefore, the reason why UA stone patients have impaired renal function remains unsolved from the present study. Despite these limitations, this is the first report to assess the implication of serum UA levels on renal impairment in patients with UA stones. In addition, the current evidences suggested the influence of MetS on UA stones and CKD. Therefore, it is necessary to improve MetS in patients with UA stones in order to prevent the progression of renal impairment.

## Conclusion

Patients with UA stones had significantly worse renal function than controls and CaOx/CaP patients regardless of hyperuricemia. Urolithiasis (CaOx/CaP and UA stone) and hyperuricemia had an association with impaired renal function. Our findings encourage clinicians to initiate intensive treatment and education approaches in patients with urolithiasis and/or hyperuricemia in order to prevent the progression of renal impairment.

## Supporting information

S1 Dataset Minimal data are available in the S1 Dataset (MS Excel file). (XLSX)
